# Why Have Detection, Understanding and Management of Kidney Hypoxic Injury Lagged behind Those for the Heart?

**DOI:** 10.3390/jcm8020267

**Published:** 2019-02-21

**Authors:** Zaid Abassi, Seymour Rosen, Simon Lamothe, Samuel N. Heyman

**Affiliations:** 1 Department of Physiology, Ruth & Bruce Rappaport Faculty of Medicine, Technion-IIT, Haifa 31096, Israel; 2 Department of Laboratory Medicine, Rambam Health Care Campus, Haifa 31096, Israel; 3 Department of Pathology, Beth Israel Deaconess Medical Center and Harvard Medical School, Boston, MA 02215, USA; srosen@bidmc.harvard.edu (S.R.); slamoth1@bidmc.harvard.edu (S.L.); 4 Department of Medicine, Hadassah Hebrew University Hospital, Mt. Scopus, Jerusalem 91240, Israel; heyman@cc.huji.ac.il

**Keywords:** acute kidney injury, myocardial infarction, ischemia, biomarkers, management, diversity outcome

## Abstract

The outcome of patients with acute myocardial infarction (AMI) has dramatically improved over recent decades, thanks to early detection and prompt interventions to restore coronary blood flow. In contrast, the prognosis of patients with hypoxic acute kidney injury (AKI) remained unchanged over the years. Delayed diagnosis of AKI is a major reason for this discrepancy, reflecting the lack of symptoms and diagnostic tools indicating at real time altered renal microcirculation, oxygenation, functional derangement and tissue injury. New tools addressing these deficiencies, such as biomarkers of tissue damage are yet far less distinctive than myocardial biomarkers and advanced functional renal imaging technologies are non-available in the clinical practice. Moreover, our understanding of pathogenic mechanisms likely suffers from conceptual errors, generated by the extensive use of the wrong animal model, namely warm ischemia and reperfusion. This model parallels mechanistically type I AMI, which properly represents the rare conditions leading to renal infarcts, whereas common scenarios leading to hypoxic AKI parallel physiologically type II AMI, with tissue hypoxic damage generated by altered oxygen supply/demand equilibrium. Better understanding the pathogenesis of hypoxic AKI and its management requires a more extensive use of models of type II-rather than type I hypoxic AKI.

## 1. Introduction

Despite the high incidence of acute kidney injury (AKI) and its association with an alarming increase in morbidity and mortality, the therapeutic approaches for AKI are still disappointing and rely mainly on supportive measures [1,2]. This deficiency stems from the poor understanding of the pathogenesis of AKI and due to the delayed detection of AKI, as this syndrome is often asymptomatic during its early stages. Concerning the latter, the diagnosis of AKI is challenging since it mainly relies on serum creatinine (Scr), which suffers from major limitations as reliable biomarker for measurement of kidney function [3]. Specifically, in the setting of AKI, the time relationship between changes in SCr and concomitant changes in GFR does not allow accurate estimation regarding timing and reversibility of renal injury and the severity of kidney dysfunction, thus delaying diagnosis and intervention [3].

As opposed to the lack of breakthroughs in the diagnosis and management of AKI, great leaps took place in the setup of acute myocardial infarction (AMI). Mortality following AMI declined dramatically over the last 40 years and post-MI morbidity and mortality improved substantially, thanks to the early detection of evolving myocardial hypoxic injury and the achievement of myocardial salvage by the prompt restoration of coronary blood flow, first with early thrombolysis, and later with immediate or delayed coronary interventions and surgery. Currently, early mortality among patients treated for acute ST-elevation MI (STEMI) is about 4%, some 80% lower than a few decades ago, underscoring the importance of early detection of AMI and of prompt intervention to restore regional blood flow.

Renal parenchymal hypoxia plays a pivotal role in various conditions leading to AKI, and a major clinical challenge is its early detection and the differentiation between hypoxic injury and other factors leading to AKI.

The aim of this review is to compare the clinical tools available for the timely detection of incipient or evolving acute hypoxic organ damage in the heart and kidney and to analyze the causes leading to the lack of advances in the diagnosis and management of hypoxic AKI, as compared with the outstanding clinical achievements in ischemic myocardial injury. This review will encompass the physiologic complexity of acute hypoxic kidney dysfunction, as compared with the heart, and as summarized in Table 1 putting side-by-side clinical features and available techniques detecting real-time organ injury. We shall further underscore what we believe are basic misconceptions regarding hypoxic AKI that have for decades diverted interventional efforts in the attempt to prevent or amend AKI in the wrong direction.

## 2. Comparing the Pathogenesis of Renal and Cardiac Hypoxic Injury

Myocardial ischemia is caused by interrupted blood supply, either as an acute coronary occlusion (caused by a ruptured plaque within an atheromatous plaque), or as a chronic narrowing of the coronary lumen, mostly due to an atheromatous plaque. The former clinical scenario leads to what is called Type I AMI, reflecting organ ischemia (i.e., anoxia due to total cessation of regional blood flow), whereas chronic narrowing of the coronary lumen leads to a transient and usually reversible regional hypoxia with an anginal syndrome (typically characterized by reversible pain), appearing whenever oxygen demand surpasses the restricted oxygen supply, for instance during exercise or tachycardia, and resolving as oxygen demand declines. If severe and protracted enough, myocardial hypoxia related to anginal syndrome may evolve into frank tissue injury, termed Type II MI [4]. The myocardium is characterized by an oxygenation gradient, with pO_2_ declining from the sub-pericardial to the innermost sub-endocardial regions, especially in regions with coronary stenosis, whenever oxygen demand/supply balance is altered: for instance when diastolic intraventricular pressure increases, undermining sub-endocardial myocardial blood flow and oxygen supply [5,6,7]. Consequently, a trans-myocardial gradient of hypoxia-induced response appears, such as enhanced expression of hypoxia-inducible factors (HIF) specifically in subendocardial regions [8], or ultimate myocardial injury, first manifested at the innermost portions of the myocardium and spreads outwards as hypoxia persists or intensifies. Intermediate degrees of sub-lethal hypoxia may invoke functional impairment, with altered myocyte contraction and relaxation, myocardial hibernation and apoptosis, whereas prolonged ischemia invokes tissue necrosis and a local inflammatory response [9,10]. Adaptive responses to chronic hypoxia such as the development of collateral microcirculation, as happens in patients with multi-vessel disease, often mitigate the outcome of acute vessel occlusion, leading to subtler tissue damage, in the form of limited subendothelial necrosis (often presented as non-Q wave myocardial infarction). By contrast, acute coronary occlusion due to ruptured plaque without prior chronic hypoxia, as occurs in young smokers with a single vessel disease, is usually manifested as STEMI, with en-block trans-mural myocardial infarction that spreads from the sub-endocardial myocardium outwards along time as ischemia persists. Rapid restoration of regional blood flow by thrombolysis, endovascular interventions (transluminal angioplasty and stenting) or with bypass operations provide myocardial salvage and may restrict the spread of necrosis. Cellular mechanisms of injury are beyond the scope of this short review but the outcome of ischemia and subsequent reperfusion in these settings are the consequence of energy store depletion and access formation of free-radicals, leading to the impairment of vital cellular functions and irreversible structural damage.

**Figure 1:**
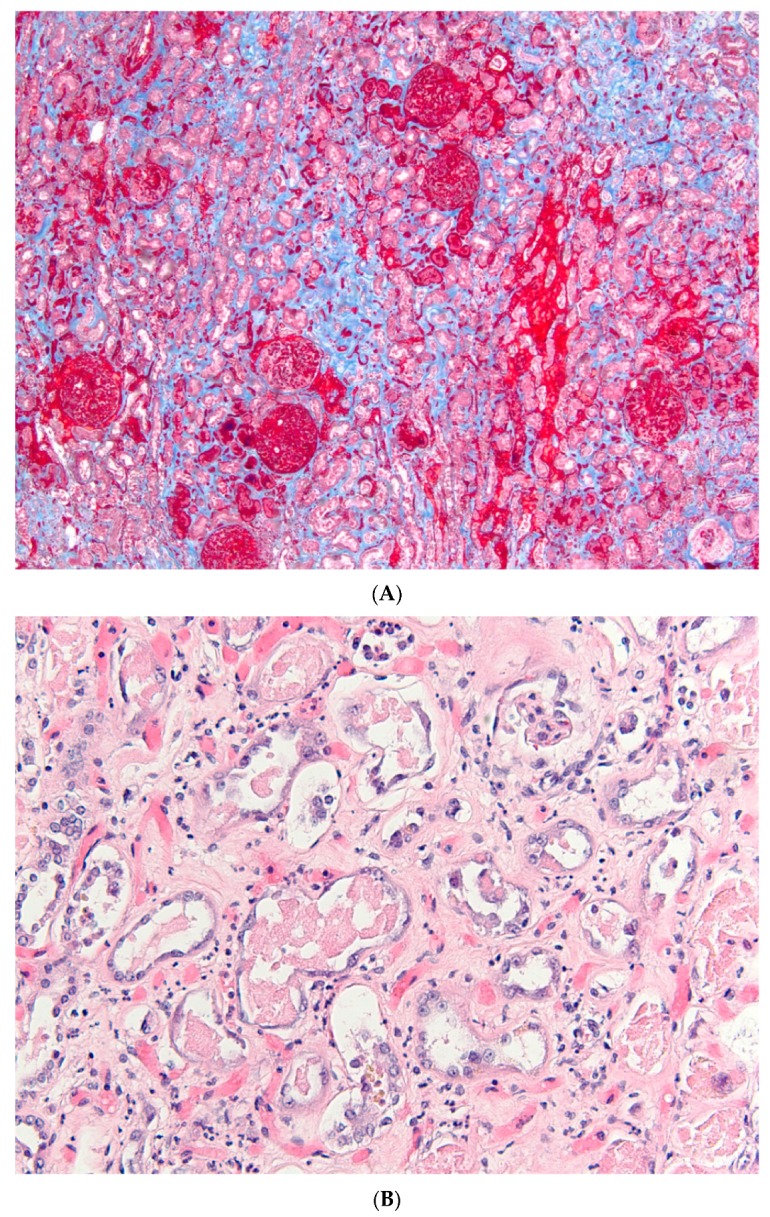
Renal cortical morphology of type I ischemic AKI. Resuscitation was initiated in a 71 years old patient with AMI who remained pulseless for an hour before the restoration of effective cardiac rhythm. The patient’s blood pressure was only briefly maintained by intravenous pressors, but he subsequently developed intractable cardiogenic shock and anuria, dying 5 days after the initial incident. Autopsy findings resemble renal morphology following prolonged WIR in rodents: In (**A**) changes of ischemic hemorrhagic necrosis are shown, superimposed on renal parenchymal tissue with fibrotic changes. Proximal tubules are illustrated in high-power photograph (**B**). The tubular epithelium is flattened and shows extensive regenerative changes and formation of casts. Scattered polymorphonuclear infiltration is also noted. (Masson’s trichrome and H&E, original magnifications ×40 and ×200, respectively).

As compared with the heart, the kidney presents with only a few uncommon clinical conditions that physiologically mimic Type I AMI with extensive ischemia and reperfusion injury. Acute main- or segmental renal arterial occlusion, for instance due to arterial emboli, is one example. Functional impairment may be subtle in these instances, reflecting enhanced filtration in remnant nephrons, unless an embolus affects a single functioning kidney, or in the presence of a-priori renal failure without functional reserve [11]. Acute global renal ischemia with AKI may develop also in kidneys harvested for transplantation or during cross-clamping of the aorta or cardiopulmonary bypass operations, where the organ is subjected to cold ischemia-reperfusion injury. Acute global warm renal ischemia with AKI may also very rarely develop due to profound and protracted shock in post-partum settings [12], or as shown in Figure 1, following prolonged pulseless cardiorespiratory resuscitation with the use of huge amounts of catecholamines [13].

**Figure 2:**
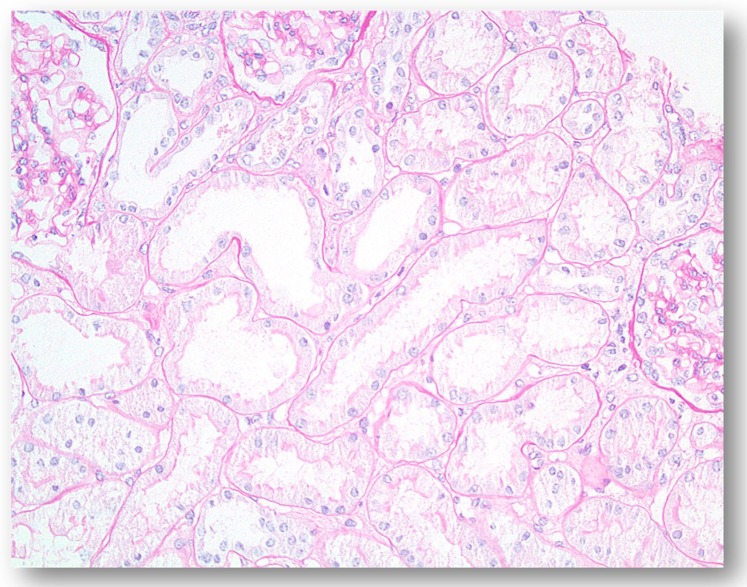
Renal cortical morphology of type II hypoxic acute kidney injury (AKI). A 18 year old male who presented with a one-day history of nausea and copious vomiting, accompanied by low-back and lower abdominal pain for which he took ibuprofen. The previous day he had consumed a fifth of Jack Daniels, smoked marijuana and played drums all night. Initial creatinine upon admission was 6.0 mg/dL with laboratory evidence of mild rhabdomyolysis (creatine kinase 2025u, normal 90–250u). Anuric AKI developed, requiring a single hemodialysis. Biopsy, obtained at peak creatinine of 14.7 mg/dL on 7th day shows maintained cortical parenchyma. (Periodic Acid-Schiff (PAS), original magnification ×200).

However, most cases of hypoxic AKI encountered in the clinical practice are not associated with total or near-total cessation of renal blood flow, and is believed to represent a Type II organ injury pattern, reflecting imbalanced regional renal oxygen supply and consumption, especially in regions which are a-priori physiologically hypoxic. As with the heart, the renal parenchymal oxygenation profile is also non-homogenous, with a cortico-medullary oxygen gradient and an inner medullary pO_2_ as low as 20 mmHg under normal physiologic conditions [14]. Physiological medullary hypoxia reflects very limited blood supply (some 10% of total renal blood flow, delivered by vasa recta), medullary oxygen shunts across descending and ascending vasa recta, and intense regional tubular transport and oxygen consumption. Medullary physiologic hypoxia is the price paid for the construction of the countercurrent urine concentrating apparatus and the very low regional blood flow required for the generation of medullary hyperosmolality. Complex mechanisms are designed to maintain safe oxygen levels in the medulla by the regulation and matching of regional blood flow and oxygen consumption for tubular transport. Prostaglandins, nitric oxide and adenosine are key regulators of these mechanisms [15]. Additional physiologic factors that affect distal tubular transport activity and oxygen consumption are glomerular filtration rate (GFR) and the extent of proximal tubular reabsorption. Enhanced GFR and diminished proximal tubular transport increase distal tubular transport workload, while reduced GFR and enhanced proximal tubular reabsorption reduce oxygen consumption by distal tubular segments and may paradoxically improve medullary oxygenation [16]. Renal oxygenation profile may diversely be affected in the cortex and medulla. For instance, controlled moderate hypotension (with reduced cortical oxygenation but maintained vasa recta blood flow) may result in declined cortical oxygenation, while medullary pO_2_ paradoxically increases as tubular transport workload diminishes. By contrast, medullary oxygenation eventually declines when mean blood pressure falls below 60–65 mmHg, as vasa recta blood flow and medullary oxygen delivery eventually decline [17]. The term “renal angina syndrome” has been proposed to describe the situation of the renal medulla, which functions on the verge of hypoxia, comparable to the myocardial anginal syndrome. Pre-renal failure exists in this setup, when mean blood pressure is kept above 60–65 mmHg, without structural damage or tubular functional defects. As medullary hypoxia intensifies with a further decline of blood pressure, tubular injury and dysfunction develops, with an activation of tubulo-glomerular feedback mechanisms that reduce GFR. Indeed, this condition of transformation from pre-renal failure to hypoxic AKI has been termed “acute renal success”, with renal parenchymal injury remaining limited and often undetected in biopsies (Figure 2) thanks to reduced GFR, as long as medullary blood supply is not critically diminished [15].

As opposed to the rare scenarios leading to total cessation of blood flow with ischemic injury, there is a host of clinical conditions leading to renal parenchymal hypoxia and to the propensity to develop hypoxic AKI due to imbalanced regional oxygen supply and consumption. Chronic kidney disease leads to renal hypoxia through rarefication of microvasculature, interstitial fibrosis and increased workload imposed on remnant nephrons [18,19]. The diabetic kidney is also hypoxic, reflecting both altered medullary flow, combined with enhanced tubular transport and oxygen requirements [20,21]. As detailed above, all conditions leading to reduced renal perfusion may affect the renal parenchymal oxygenation profile. Administration of hypertonic saline [22], mannitol [23] or SGLT2 inhibitors [24,25] increase distal solute delivery and may intensify transport workload and medullary hypoxia. Inhibition of prostaglandins synthesis with non-steroidal anti-inflammatory agents or altered nitric oxide production invokes profound medullary hypoxia by both the selective reduction of vasa recta blood flow and augmentation of sodium transport in medullary thick ascending limbs (mTALs) [26]. Likewise, iodinated contrast agents cause intense medullary hypoxia by reducing vasa recta flow and enhancing tubular transport [27]. Other nephrotoxins may induce hypoxic injury by the reduction of regional blood flow alone (cyclosporine, heme-containing molecules) or in association with enhancement of tubular transport (amphotericin) [26]. Near-drowning in seawater is an archetype of renal oxygenation imbalance, due to both reduced renal oxygen supply (because of systemic hypoxemia, intense sympathetic activity and perhaps a transient hypotension) and enhanced oxygen consumption (with large amounts of absorbed hypertonic saline profoundly enhancing solute delivery for re-uptake in the distal nephron) [28]. Worth noting is that we often induce predictable iatrogenic type II hypoxic AKI in justified clinical settings such as coronary interventions using iodinated contrast media in very high-risk patients with cardiogenic shock. Yet, understanding this disorder might reduce it by measures aimed to maintain renal oxygen balance, as outlined in Future Challenges.

Unlike the homogenous myocardium, renal morphology is highly complex and tubular segments and other parenchymal components differ in their ability to cope with hypoxic stress. The proximal tubule is highly susceptible to hypoxic injury since it cannot tolerate prolonged anaerobic glycolysis. By contrast, mTALs are capable to endure prolonged hypoxia as long as transport activity stops, but rapidly develop hypoxic damage in proportion to oxygen consumption for ion transport [29,30]. Transport activity also intensifies hypoxic injury to S3 segments [31], while collecting ducts are highly resistant to hypoxia and are able to express large amounts of HIF in response to intensified ambient hypoxia [32]. In the outer medulla, seemingly tubular segments compete with each other on sparse oxygen availability, and excess tubular transport in on segment intensify damage in others [33]. A further complicating factor is the often complex and multifactorial nature of renal parenchymal injury and dysfunction, with renal hypoxic injury act in concert with direct tubular toxicity (as with amphothericin, or in hemo/myoglobinuric renal failure), or with altered renal hemodynamics (for instance in sepsis) [26]. It is, therefore, often impossible to assess the independent impact of hypoxia on AKI in these settings.

In conclusion, renal ischemia, i.e., total cessation of renal blood flow is a rare form of hypoxic AKI that resembles Type I AMI, with tissue ischemia and reperfusion injury. By contrast, in most cases, renal parenchymal- and particularly medullary hypoxic injury may develop whenever oxygen supply/demand balance is disrupted, resembling pathophysiologically Type II AMI in patients with fixed stenotic coronary arteries [4]. We propose using the terminology of type I and type II hypoxic AKI, for these two different conditions, respectively.

## 3. Conceptual Errors in the Understanding of Hypoxic AKI

For decades, renal warm ischemia-reperfusion has been adopted by nephrologists as the representative of hypoxic AKI. Extensive research and large budgets have been invested in studying rodent models of ischemia generated by arterial cross-clamping of the renal artery for 30 and up to 60 min in mice and rats, respectively, followed by reperfusion. These models of ischemia and reperfusion (type I hypoxic AKI) parallel animal models of Type I AMI. Morphologic characteristics of tissue injury in these AKI models are ischemic time-dependent extensive proximal tubular injury predominantly in the outer stripe of the outer medulla, accompanied by congestion and substantial inflammation [34], with renal functional impairment grossly proportional to the extent of injury. Medullary thick limb injury in these models does not occur since tubular transport ceases upon cross-clamping, and is low during reperfusion, likely because of reduced GFR and downstream solute delivery [35]. Renal morphology in these models resembles en-bloc necrosis and inflammation, noted in the uncommon clinical cases of global renal ischemia, as shown in Figure 1.

As outlined above, this injury mechanism with global organ ischemia does not physiologically mimic type II hypoxic AKI, where near-complete cessation of renal blood flow does not occur. Over the years models of hypoxic AKI were constructed, both in isolated perfused kidneys and in intact animals in vivo, based on medullary oxygen imbalance, where renal oxygen supply and tubular transport persist throughout the induction of injury [36,37]. Tubular hypoxic stress and injury in these models is principally noted in mTALs and to a lesser extent in S3 segments in the outer medulla, ranging from stabilization of HIF signals, through apoptosis or reversible phases of cell injury (nuclear pyknosis, mitochondrial swelling) to frank necrosis with cell membrane disruption. Injury gradient is evident, most prominent in the inner layers of the outer medulla and in the interbundle zone, most remote from vasa recta and oxygen supply [32,38,39]. In these models tissue injury may be focal, without prominent inflammation, and with functional impairment (reduced GFR) often out-of-proportion to the very limited focal tubular injury. We believe that such models are physiologically relevant to type II hypoxic AKI, with limited tubular injury as in Figure 2. Using the warm ischemia-reperfusion AKI models for years has been a formidable obstacle in the development of clinically relevant therapeutic strategies, treating animals with an artificial disease that is irrelevant to the human scenario [36,40].

## 4. Clinical Presentation of Hypoxic Cardiac vs. Renal Injury

Myocardial ischemia is most often manifested immediately with chest pain and accompanying complains, such as radiating pain, nausea and vomiting, cold perspiration, shortness of breath and doom feeling. Angina pectoris is initiated or aggravated by physical activity and enhanced myocardial oxygen demand. Yet, some patients, especially elderly and diabetic individuals might undergo painless myocardial ischemia or hypoxia due to altered sensorium.

As opposed to protean overt symptoms in most cases of myocardial injury, clinical symptoms in the clinical settings of hypoxic AKI are most often absent, likely since tissue injury in subtle and focal, and out of proportion to the extent of functional impairment. Rare exceptions are type I hypoxic injury due to acute total or segmental renal infarction that may be manifested as flank pain mimicking renal colic. Gradual renal artery stenosis may be revealed among patients during the evaluation of secondary hypertension, and symptoms of severe hypertension or pulmonary congestion may develop in the case of renal arterial occlusion of a single functioning kidney or in the rare cases of bilateral renal ischemia.

## 5. Detection and Assessment of Myocardial Hypoxic Injury

A large and easily accessible arsenal of diagnostic tools is available in the clinical practice for the immediate detection of myocardial hypoxia. Myocardial ischemia can be detected in real time as it evolves by typical electrocardiographic changes and by altered regional wall motion, easily sensed by echocardiography, which also helps ruling out major differential diagnoses. Dynamics in these parameters can be assessed in type II ischemia during enhanced workload and oxygen consumption, with exercise test or its equivalents. Myocardial perfusion can be identified by radiolabled scans and by MRI, and tissue injury can be validated by the detection of released myocardial constituents, such as troponin isoforms, myoglobin and others. The extent of myocardial necrosis grossly correlates with the rise in these biomarkers of cardiac muscle injury. With all these technologies myocardial hypoxia or ischemia is usually easily detected and intravascular lesions can be located and managed by non-invasive and invasive coronary imaging and interventions.

Altogether, cardiologists possess reliable and readily available diagnostic tools that provide immediate assessment of cardiac function and indices of tissue injury, studies that can be performed repeatedly with grossly clear-cut threshold definitions of myocyte injury.

## 6. Detection of Hypoxic Renal Injury

Delayed involvement of nephrologists is an important obstacle in achieving real-time identification, appropriate management and improved outcome of AKI. Furthermore, comparable tools for the prompt and timely diagnosis of AKI in general and specifically of hypoxic renal injury are unfortunately lacking. The parameters used by AKIN or RIFLE criteria for grading of AKI stages [41,42], namely the rates of urine output and rise in plasma creatinine, are non-specific and non-immediate upon injury. The clinical scenario and the presence of orthostatic hypotension may suggest pre-renal failure, and renal Doppler-ultrasound can rule out post-renal causes and assess renal vascular resistance, and may identify features of chronic renal disease. Urinalysis and microscopy may help detecting glomerulopathies and might identify typical features of acute tubular necrosis or crystal nephropathy. Analysis of urine osmolality, sodium and creatinine, and their corresponding plasma values further may help differing pre-renal failure from tubular injury. Yet, often results are within a very wide gray zone with low specificity, and these parameters are markedly affected by the use of fluids and diuretics and in the presence of pre-existing renal disease. Enhancement imaging with iodine-containing- or gadolinium-based contrast agents is associated with the risk of further renal injury or nephrogenic systemic fibrosis, respectively.

Novel technologies currently under development provide non-enhanced imaging of renal morphology, function, microcirculation and oxygenation [43]. Additional promising diagnostic tools are biomarkers of renal tissue injury [44]. The potential use of these two advanced tools in the early detection of AKI in general and in are briefly discussed below.

## 7. Biomarkers of Acute Kidney Injury

The great development in molecular biology during recent decades has led to a major advance in the search for novel biomarkers for early detection of AKI [45], as evident by the discovery of several key biomarkers of myocardial injury with various specificity and sensitivity, including neutrophil gelatinase-associated lipocalin (NGAL), kidney injury molecule-1 (KIM-1), liver-type fatty acid-binding protein (L-FABP), netrin-1 and IL18 [45,46,47,48]. NGAL is one of the most prominently up-regulated genes in the kidney after AKI, particularly in distal nephron segments and damaged renal tubule [45,46]. This biomarker is increased in urine and plasma early after renal ischemia in mouse and rat models [49], and following cardiopulmonary bypass, radiocontrast administration, sepsis, and kidney transplantation, specifically in patients who subsequently developed AKI [50]. Meta-analysis of data from 19 studies, which included more than 2500 patients, revealed that rises in serum or urine NGAL levels are diagnostic for AKI [51]. As opposed to NGAL, KIM-1 is a biomarker for proximal tubular injury, and is a hallmark of toxic and ischemic proximal tubular injury [46,47]. Double labeling immunohistochemistry in various experimental and human renal diseases revealed that KIM-1-positive tubules are associated with macrophages and areas with increased expression of α-smooth muscle actin (α-SMA), a marker of myofibroblast transformation [46,52,53]. Interleukin 18 (IL-18), a pro-inflammatory cytokine belonging to the IL-1 superfamily has been reported to mediate experimental ischemic proximal tubular injury and its accompanied pro-inflammatory responses [54]. IL-18 is produced by phagocytes attracted to injured proximal tubules, activated by caspase 1, and excreted into the urine upon renal ischemic injury [54]. Although IL-18 was found to be a helpful biomarker for early detection of AKI, it is less sensitive than NGAL [55,56]. Most recently additional two cycle arrest biomarkers were added to the growing list of AKI biomarkers, namely, insulin-like growth factor-binding protein 7 (IGFBP7) and tissue inhibitor of metalloproteinases-2 (TIMP-2), with the potential to provide new mechanistic insights into the pathogenesis of AKI on one hand and the ability for early detection of this clinical setting, on the other [48,57].

Despite the expectations from these novel biomarkers to be sensitive, practical, and accurate in predicting AKI, the results were not unambiguous. Indeed, some of these biomarkers show unequivocal ability to predict AKI in certain settings as mentioned above. However, many of them suffer from drawbacks mainly due to broad range of its predictive accuracy. Moreover, the circulatory concentration profile of most biomarkers is adversely affected by the fluid balance and diuretic therapy. Though this concern regarding urinary biomarkers could be overcome by correcting their excretion to urinary creatinine, in certain clinical settings the creatinine kinetics is dramatically altered as the case in critical ill patients where most of the AKI cases are witnessed. Additional concern is the fact the most of the tested biomarkers are present in the circulation and their excretion in the urine does not necessarily reflect renal origin/damage. For instance, NGAL is expressed by several cell types, including endothelial cells, smooth muscle cells and macrophages in atherosclerotic plaques, where it assumedly plays a role in the pathogenesis of atherosclerosis, endothelial dysfunction, inflammatory processes and extracellular matrix remodeling [58]. Moreover, since the urinary origin of the various biomarkers are cell-specific, and AKI could be due various aetiologies, i.e., ischemia, nephrotoxicity, inflammation and renal outflow obstruction, with differential involvement of cell types, it is unlikely that a single biomarker would predict AKI reliably, the mechanisms underlying AKI or the identity of the damaged renal cells: proximal, distal, collecting duct epithelial cells, or interstitial cells [48]. Support for the use of combined biomarkers to improve their predictability value came from a study in adult patients who underwent CPB surgery, where using urinary biomarkers KIM-1, NAG, and NGAL predicted AKI 3 h from the operation by 0.65 for KIM-1; 0.63 for NAG; and 0.65 for NGAL [59]. When two biomarker was applied, the sensitivity of AKI prediction 3 h from surgery was enhanced to 0.78 [60], and escalated even to a higher specificity (0.94) when four biomarkers were used [59]. Finally, the specificity and sensitivity of most of these biomarkers are deleteriously affected by confounding factors as the presence background diseases such as CKD, cancer, heart failure, atherosclerosis or inflammation, which may cause false positive diagnosis [45]. This may explain the high specificity and sensitivity of the initial results where the reliability of theses biomarkers were tested in children who underwent CPB or radiocontrast administration [45,46], as compared with the lower accuracy when the studies were repeated in adult patients (usually with background diseases) who underwent similar clinical procedures [48].

In sum, the AKI biomarkers field is evolving. Despite their advantage as noninvasive and reasonable specificity for renal injury, more comprehensive studies are still requested to determine their clinical application.

## 8. Renal Functional Imaging

Although determination of vital organs function and dysfunction still largely relies on biochemical analysis of blood/serum and/or urine, imaging of these organs is evolving rapidly and holds great promise for improving detection of structural, functional and even molecular changes in these tissues [61]. The kidney is of special interest yet challenging, as it has unique anatomic and ultrastructural features. Specifically, the multi functions of the kidney require different types of epithelial cells, regional blood flow and oxygen consumption along the nephron and the cortical and medullary tissues. Therefore, development of functional imaging is crucial for diagnosis of physiological and pathophysiological alterations in the kidney.

Historically, renal perfusion and oxygenation under various conditions was determined either invasively via laser-Doppler flowmeter and oxygen microelectrodes [17,23,33] or indirectly via measurement of urinary pO_2_ [22]. Subsequently, imaging of renal perfusion and GFR were developed, with arterial spin labeling (ASL), an MRI methodology applied to determine total RBF [62]. ASL uses magnetically tagged water with a radiofrequency pulse. Briefly, a control image is obtained without contrast, followed by a radiofrequency pulse of the blood before it enters the kidney. The labeled image is then subtracted from the baseline image, generating a map of the signal difference created by the perfused blood [63]. Although initially ASL was not considered sensitive, upgrading imaging acquisition techniques improved its performance, where it can currently discriminate between medulla and cortex according to meta-analysis of 53 studies [64]. For instance, ASL allows detecting early renal damage following cardiopulmonary bypass procedures, where the incidence of AKI may reach up to one third of patients [50,65]. As cardiopulmonary bypass-induced AKI was associated with renal hypoperfusion and vasoconstriction, ASL was sensitive enough to detect reduced RBF and GFR [49]. This methodology was found to be valid in kidney-transplanted patients with delayed graft function [66]. Similarly, ASL exhibited high sensitivity in detecting impaired regional blood perfusion along kidney injury and histological changes in correlation with the severity of the renal insult in experimental models of AKI induced by ischemic/reperfusion for 35–45 min [67]. Moreover, ASL detected long term outcome of renal remodeling and hypo perfusion/remodeling or full recovery a few weeks after the AKI induction [61,67].

In the last two decades, blood oxygen level-dependent (BOLD) MRI and related technologies provided the most striking advance in non-invasive measurement of intra renal oxygenation profile in humans and in experimental animals [61,68]. Bold MRI uses the different properties of oxygenated and non-oxygenated hemoglobin, where the latter is magnetic, to determine tissue oxygen levels [61]. Several studies have shown that R2* relaxation rate is inversely correlated with tissue pO_2_ and can be mapped throughout BOLD MRI of the kidneys [69,70,71]. Specifically, using BOLD MRI revealed that administration of furosemide to normal subjects significantly decreased outer medullary R2* [72], indicating improved oxygenation, in agreement with findings with oxygen microelectrodes [23]. Yet it did not cause such reduction in patients with CKD and among hypertensive subjects, although basal R2* was comparable in the three studied groups [72]. Despite the increasing popularity of this convenient method, there are several confounding factors that may adversely affect its reliability, such as fluid balance, salt intake, smoking, and pulmonary function [69]. Respiratory gating is also required to overcome kidney motion-related artifacts. Dynamic contrast-enhanced (DCE) MRI is an additional imaging approach aimed at measuring GFR and renal blood flow, using gadolinium-based contrast agents [73,74]. For this purpose, serial images of the kidney are obtained as the contrast is filtered and used to calculate GFR. The DCE MRI method was found to be superior to other MRI techniques that use radionuclides for the measurements of GFR [75]. Finally, hemodynamic response imaging (HRI), a non-enhanced functional MRI method, provides insights regarding renal perfusion and vascular reactivity in response to alternating hypercapnia and hyperoxia [76,77].

Sodium MRI provides an additional unique capability of the assessment of outer medullary function. In healthy state and especially in pre-renal impairment, this gradient reflects the efficient concentrating mechanism generated in part by avid sodium transport across mTALs. Disruption of the normal cortico-medullary sodium gradient that appears within a few hours following the disruption of medullary oxygenation balance is a very early indicator of medullary hypoxic injury, when morphologic indices of tubular damage are still very focal and perhaps reversible, and plasma creatinine hardly rises [78]. This finding likely goes together with increased fractional sodium excretion and the transformation from highly concentrated urine to isosthenuria [79]. Thus, renal sodium MRI might provide the equivalent of the detection of compromised myocardium with segmental wall-motion disturbances by stress-echocardiography.

These wonderful non-invasive tools alone or in combination provide tremendous insight about real-time changes in renal structure, hemodynamics, glomerular filtration, oxygenation and function. Unfortunately, while they may serve as advanced experimental probes, their clinical use is limitted, as they are not available in most institutes. Furthermore, most patients requiring diagnostic evaluation of AKI are in critical care settings, connected to monitoring and life-maintaining equipment that preclude bedside usage of MRI. A more feasible and promising technology for the early real-time detection of evolving medullary hypoxia and for assessing the risk of hypoxic AKI is a continuous determination of urinary pO_2_ at the catheter tip, as recently shown in the settings of cardiac surgery [80].

Imaging that enables the detection of injured tissues is another exciting plausible technology to be studied [81]. Focal tubular apoptosis and necrosis were traced by a specific small molecule in evolving hypoxic and septic models of AKI [82]. Radiolabeling of this molecule enables detection by PET, and has been used to spot apoptotic malignant cells in response to chemotherapy or the development of neuronal damage. However, the renal clearance of this compound currently precludes its use in the assessment of tissue damage in AKI. In that respect, recently introduced advanced Doppler ultrasound technologies provide fine assessment of the renal microcirculation at the bedside [83] and may be clinically more applicable.

## 9. Future Challenges

Current technologies enable the precise mapping of hypoxic regions (such as BOLD MRI in vivo, or immunostaining for HIF isoforms or for pimonidazole adducts in kidney samples) and the detection of renal parenchymal injury in animal models with biomarkers. To better appreciate the pathophysiology and the impact of interventions, such endpoints should be included in experimental settings in whole animal studies, in parallel with precise morphology, since looking only at parameters of glomerular filtration, or even of tubular function may not suffice. For instance, the use of loop diuretics in clinical trials for the prevention of hypoxic AKI induced by radiocontrast agents has been abandoned as plasma creatinine increased [84]. Likely, the rise in creatinine reflected insufficient volume replacement and pre-renal failure [85], while in fact medullary oxygenation and tubular viability have been improved [38,86]. In later clinical trials in high-risk patients, large fluid replacements combined with furosemide attenuated the risk of radiocontrast-induced nephropathy [87]. Most importantly, the correlation of urine biomarker levels with the extent and distribution of tubular injury has not been studied in depth. As mentioned above, some urine biomarkers, such as KIM-1 are generated by proximal tubular segments while others, such as NGAL originate from distal segments. Precise quantitation of injury at specified nephron segments at various locations within the renal parenchyma, as well as the extent of chronic fibrotic changes and the renal distribution of hypoxia (with pimonidazole) and hypoxia-response (HIF detection) can be achieved in perfusion-fixed thin section preparations in experimental models of AKI [88,89]. It is about time to try and correlate the extent of tubular injury at different segments with their corresponding cell-specific biomarkers in order to validate the sensitivity and specificity of these biomarkers in animal models of acute- and acute on chronic AKI. Such studies may improve our understanding of human AKI and may direct us in the generation of appropriate panels of biomarkers to appropriate for varied clinical conditions, for instance biomarkers for proximal tubular injury for suspected aminoglycoside nephrotoxicity, or a panel of distal tubular biomarkers following near drowning, or in AKI following the administration of SGLT2 antagonists.

Most non-invasive functional imaging techniques are not applicable for patients in critical care settings, and their use is restricted to animal studies and to human studies under ambulatory settings. Further development is needed of technologies that will enable bedside mapping of renal oxygenation profile and of real-time renal functional parameters.

## 10. Conclusions

The detection, prevention and successful treatment of hypoxic AKI have only moderately been advanced over recent decades, as compared to the substantial achievements in the diagnosis and management of ischemic heart disease. Disorders of renal parenchymal oxygenation are much more compound than those of the heart muscle. Furthermore, nephrology lags far behind cardiology in the diagnosis and management of hypoxic injury because of the lack of clinical symptoms and overt early physiologic signs, and the absence of diagnostic tools that provide immediate and highly specific results during the evolution of kidney injury. Changes in urine volume and the pattern of the rise in creatinine are non-specific and often retarded crude indices of renal dysfunction. Novel biomarkers provide new tools for the early detection of renal parenchymal injury, yet, the heterogeneity of damage patterns affecting different tubular segments in various toxic and hypoxic insults may differently affect some segment-specific biomarkers. There is no available human data regarding the correlation of biomarker levels and tissue injury extent and pattern, and such data should be evaluated in animal studies. The specificity and sensitivity of biomarkers in the detection of acute-on-chronic renal injury need improvement. Functional imaging is a fantastic experimental tool, but its availability and potential use in the clinical practice are currently limited.

The pathogenesis of AKI is often complex, with direct nephrotoxicity, hypoxia, altered microcirculation and other factors playing in concert. Differentiation between the causative factors is therefore principally based on clinical judgment and the assessment of the individual contribution of hypoxia may be based only on hypoxia mapping by functional imaging or by the determination of urine pO_2_.

Differing between type I and type II organ injury pattern should be based principally on the clinical scenario, both for the heart and kidney. Yet, this distinction, easily confirmed by angiography in AMI, might not be possible to establish unequivocally for AKI.

Finally, nephrologists lag behind cardiologists regarding hypoxic AKI also because of conceptual errors. Research projects have failed to distinguish between conditions with protracted renal warm ischemia and reperfusion, and those with maintained renal blood flow and transport activity but imbalanced oxygen supply and expenditure. We propose to differently address these conditions as type I and type II hypoxic AKI, terms physiologically parallel to type I and type II AMI. Whereas type I AKI is infrequent and rarely presents with renal functional impairment, most research studies use type I ischemic AKI animal models, which bear little relevance to the far more frequent conditions leading to type II hypoxic AKI. Better understanding and advances in the management of hypoxic AKI will require more studies using animal models of type II-, rather than type I renal parenchymal hypoxic injury.

## Figures and Tables

**Table 1:** Types of acute kidney injury (AKI) and acute myocardial infarction (AMI)-similarities and differences.

	Type I Ischemic AKI	Type II Hypoxic AKI	Type I AMI	Type II AMI
**Pathophysiology**	Ischemia and reperfusion	Altered oxygen supply/demand balance	Ischemia and reperfusion	Altered oxygen supply/demand balance
**Tissue blood supply**	ceases	Maintained or reduced	ceases	Maintained or reduced
**Workload and oxygen consumption**	ceases	continued/enhanced	Rapidly declines	enhanced
**Clinical scenario**	Rare Renal infarct	Common Hypotension, CKD, diabetes, NSAIDs, iodinated contrast media, osmotic dieresis etc.	AMI Due to ruptured plaque	Increased workload in the presence of coronary stenosis (hypotension, anemia, tachycardia etc.)
**Symptoms**	Flank pain, hematuria	None related to AKI	Chest pain and accompanying complaints	Chest pain and accompanying complaints
**Immediate evidence of functional impairment**	None if unilateral/segmental	None	Occasionally evidence of impaired circulation and heart failure	Occasionally evidence of impaired circulation and heart failure
**Rapid functional diagnostic tools**	None	None	Echocardiography (ECG)	Echocardiography (ECG)
**Specific biomarkers**	Available and sensitive, limited specificity †	Available, limited specificity and sensitivity †	Highly specific and sensitive in detecting injury	Highly specific and sensitive in detecting injury

† Specificity is limited especially in the presence of pre-existing renal disease and in aged patients. Tubular segment-type-specificity of the various biomarkers require panel assays. CKD: Chronic kidney diseases; NSAIDs: Non-steroidal anti-inflammatory drugs.
